# *Lactobacillus* species isolated from vaginal secretions of healthy and bacterial vaginosis-intermediate Mexican women: a prospective study

**DOI:** 10.1186/1471-2334-13-189

**Published:** 2013-04-26

**Authors:** Marcos Daniel Martínez-Peña, Graciela Castro-Escarpulli, Ma Guadalupe Aguilera-Arreola

**Affiliations:** 1 Laboratorio de Bacteriología Médica, Departamento de Microbiología, ENCB-IPN, México D.F., Mexico; 2 Laboratorio de Recursos Genéticos Microbianos, Centro Nacional de Recursos Genéticos, INIFAP, Tepatitlán de Morelos, Jalisco, Mexico

**Keywords:** Lactobacilli, Mexican population, 16S rRNA, Species identification, Vaginal microbiota, Bacterial vaginosis

## Abstract

**Background:**

*Lactobacillus jensenii*, *L. iners*, *L. crispatus* and *L. gasseri* are the most frequently occurring lactobacilli in the vagina. However, the native species vary widely according to the studied population. The present study was performed to genetically determine the identity of *Lactobacillus* strains present in the vaginal discharge of healthy and bacterial vaginosis (BV) intermediate Mexican women.

**Methods:**

In a prospective study, 31 strains preliminarily identified as *Lactobacillus* species were isolated from 21 samples collected from 105 non-pregnant Mexican women. The samples were classified into groups according to the Nugent score criteria proposed for detection of BV: normal (N), intermediate (I) and bacterial vaginosis (BV). We examined the isolates using culture-based methods as well as molecular analysis of the V1–V3 regions of the 16S rRNA gene. Enterobacterial repetitive intergenic consensus (ERIC) sequence analysis was performed to reject clones.

**Results:**

Clinical isolates (25/31) were classified into four groups based on sequencing and analysis of the 16S rRNA gene: *L. acidophilus* (14/25), *L. reuteri* (6/25), *L. casei* (4/25) and *L. buchneri* (1/25). The remaining six isolates were presumptively identified as *Enterococcus* species. Within the *L. acidophilus* group, *L. gasseri* was the most frequently isolated species, followed by *L. jensenii* and *L. crispatus*. *L. fermentum*, *L. rhamnosus* and *L. brevis* were also isolated, and were placed in the *L. reuteri*, *L. casei* and *L. buchneri* groups, respectively. ERIC profile analysis showed intraspecific variability amongst the *L. gasseri* and *L. fermentum* species.

**Conclusions:**

These findings agree with previous studies showing that *L. crispatus*, *L. gasseri* and *L. jensenii* are consistently present in the healthy vaginal ecosystem. Additional species or phylotypes were detected in the vaginal microbiota of the non-pregnant Mexican (Hispanic-mestizo) population, and thus, these results further our understanding of vaginal lactobacilli colonisation and richness in this particular population.

## Background

The vaginal ecosystem is dynamic and contains microbiota that are protective against invading pathogens, including those causing urinary tract infections and sexually transmitted infections (STIs). Lactobacilli are the best known bacteria of the normal vaginal microbiota. Their ability to produce lactic acid, H_2_O_2_ and bacteriocins makes them prime candidates for the surveillance of vaginal health because these substances are unfavourable to many other bacterial species [1,2]. However, the presence of lactobacilli may not always be beneficial because certain lactobacilli do not contribute to vaginal wellbeing [1]. In general, a *Lactobacillus*-deficient condition, characterised by an overgrowth of anaerobes, is associated with the development of numerous infectious, such as bacterial vaginosis (BV) and aerobic vaginitis, and promotes the transmission of sexually transmitted diseases, including gonorrhoea, chlamydia, syphilis, trichomoniasis, human immunodeficiency virus (HIV) and human papillomavirus (HPV) [3-5]. *Lactobacillus jensenii*, *L. gasseri*, *L. iners* and *L. crispatus* are the most frequently isolated *Lactobacillus* species from the vagina, although the native species and their relative abundance vary widely depending on the studied population [3,6,7].

In Mexican women, an early phenotypic study suggested that *L. acidophilus* was the predominant species [8], whereas *L. brevis, L. crispatus, L. fermentii* and *L. jensenii* were identified in a more recent study using carbohydrate profiling [2]. Both of these studies used the vaginal discharge of healthy women. In contrast, when genetic approaches were used, *L. acidophilus*, *L. iners*, *L. gasseri* and *L. delbrueckii* were reported to be the most prevalent species in pregnant Mexican women [9]. To our knowledge, *Lactobacillus* species found in the vaginal discharge of healthy, non-pregnant Mexican women have not been studied using genetic approaches.

The unreliability of classical identification methods, which employed sugar fermentation and other phenotypic assays, previously hindered the identification of the predominant *Lactobacillus* species colonising the vagina. The inclusion of molecular tools for species identification is extremely important because previous studies have demonstrated that strains can be incorrectly classified when using the API 50 CHL identification system alone [10,11]. Thus, microbiological and biochemical methods may be unreliable for the identification of lactic acid bacteria because of the low discriminatory power of the tests, and the intragenic diversity of *Lactobacillus* strains [12]. Therefore, the accurate characterisation of isolated strains may be achieved using a polyphasic approach, including a combination of classical culture-dependent and culture-independent phenotyping methods, along with molecular procedures [13,14]. Randomly amplified polymorphic DNA (RAPD) analysis, ribotyping and intergenic spacer PCR (ITS-PCR) analysis are commonly employed molecular methods for strain identification [10,12,15,16]. However, 16S ribosomal RNA sequence analysis is the most popular approach for classifying *Lactobacillus* strains. Analysis of 16S rRNA gene fragments is one the most powerful molecular tools for determining phylogenetic relationships among bacteria. In particular, 16S rRNA sequencing is useful in identifying bacteria that are difficult to classify by conventional methods.

Culture-dependent and culture-independent studies have found that *L. jensenii*, *L. iners*, *L. crispatus* and *L. gasseri* are the most common *Lactobacillus* species present in the vagina [17,18]. This study was performed to determine the genetic identity of *Lactobacillus* strains present in the vaginal discharge of healthy and bacterial vaginosis intermediate Mexican women.

## Methods

### Sampling procedure and bacterial strains

This study was performed with approval from the ethics committee of the National School of Biology Science (ENCB) at the National Polytechnic Institute (IPN), Mexico City. Written informed consent was obtained from all study participants. In total, 105 vaginal samples (vaginal exudates) to be used for culturing of *Lactobacillus* species were collected from 105 participating women at the ENCB. The women were between 18 and 65 years old. Exclusion criteria included pregnancy, antibiotic treatment within one month prior to sampling and sexual intercourse within three days prior to sampling. Demographic, behavioural and clinical analyses of the results from the survey of the enrolled participants have been published [19]. All samples were taken from the posterior zone of the fornix of the vagina using a sterile swab. The swab was placed in a tube containing Stuart broth (Difco, Detroit, MI, USA) and inoculated onto Man Rogosa Sharpe (MRS) agar plates (Oxoid, Basingstoke, Hampshire, England). A thin smear was examined by Gram staining and interpreted using Nugent’s criteria. Three *Lactobacillus* reference strains from the ENCB culture collection were included, and characterised in parallel with the clinical isolates. *Aeromonas caviae* strain IIH147 was used as a positive control for the enterobacterial repetitive intergenic consensus (ERIC) sequence PCR assay.

### Sample groups

**Figure 1:**
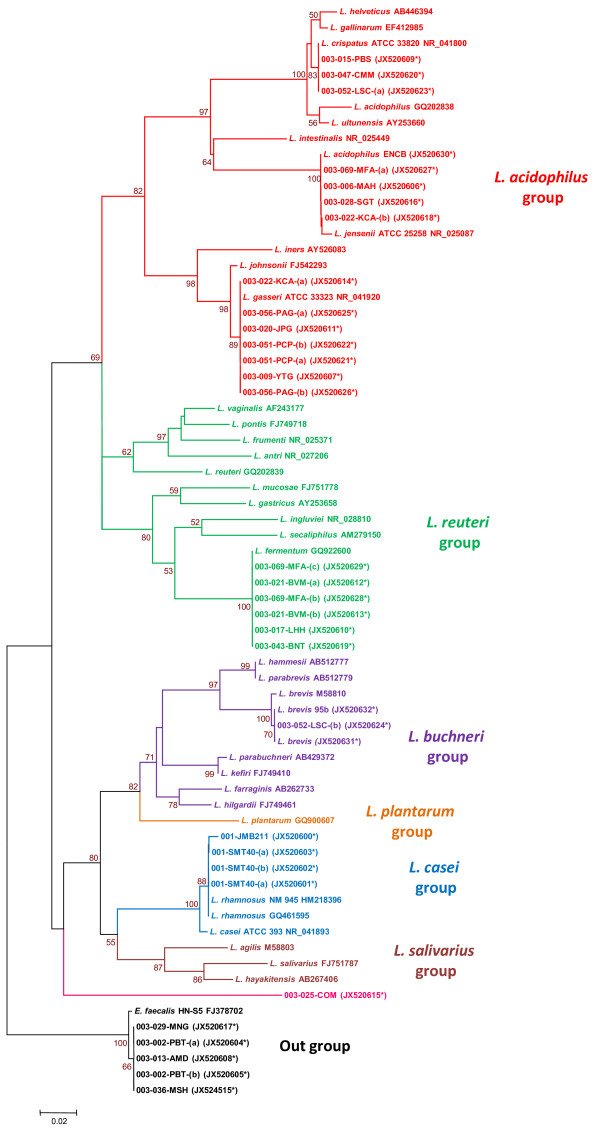
**Dendrogram based on the 16S rRNA sequences of *Lactobacillus *strains identified in this study.** The tree was generated using the neighbour-joining method. The branch lengths are proportional to the genetic distance, and the numbers shown at the branch points indicate the bootstrap values. The data set was subjected to 1,000 bootstrap replicates. The reference sequences were obtained from the GenBank database. Sequences from the clinical samples are indicated by an asterisk, and the sequence accession numbers are in parentheses (GenBank JX520600–JX520632).

The samples were classified into groups according to the Nugent score criteria proposed for normal (N), intermediate (I) and bacterial vaginosis (BV) [20]. If the Nugent score was normal, and if no other bacterial disease was found, the sample was categorised as healthy (H). In contrast, if the Nugent score was intermediate or BV and other bacteria were present, the sample was classified as unhealthy (UH) (Figure 1). The scores were categorised as follows: 0–3 was considered normal or negative for BV; 4–6 was considered intermediate; ≥7 was considered indicative of BV.

### Strain isolation

The swabs were inoculated onto MRS agar plates (Difco) and incubated at 37°C with partial CO_2 _tension for 48 h. Growth on MRS was observed for 21 of the 105 analysed samples, and one or more colonies were selected and picked for each of the 21 samples. All colonies were examined by Gram staining and catalase and oxidase tests. A total of 31 strains that were phenotypically consistent with the *Lactobacillus* genus were chosen and preserved in 25% MRS glycerol at −70°C until DNA extraction.

### DNA extraction

The isolated *Lactobacillus* strains were subcultured on MRS agar at 37°C for 48 h. The bacterial colonies were added to 500 μL of Tris-EDTA 50/20 (TE) buffer (pH 8) (Sigma-Aldrich, Saint Louis, MO, USA) and then mixed and centrifuged at 8000× *g* for 2 min. The resulting supernatant was removed by decantation and discarded, and then 175 μL of TE buffer and 10 μL of RNase A (Sigma-Aldrich) were added to resuspend the cell pellet. The sample was then incubated at room temperature for 10 min. The cells were lysed by the addition of 20 μL of 10% sodium dodecyl sulphate (SDS) (Sigma-Aldrich) and 5 μL of a proteinase K solution (20 mg/mL) (Sigma-Aldrich), followed by a 2 h incubation at 56°C. Subsequently, 20 μL of 5 M NaCl (Sigma-Aldrich) and 500 μL of TE 10/1 (pH 8) were added to the samples. The lysate was extracted once with 500 μL of equilibrated phenol, which was mixed for 10 min and then centrifuged at 8000× *g* for 5 min at 4°C. The supernatant was removed, and the aqueous phase was extracted once more with 500 μL of chloroform:isoamyl alcohol (24:1) and centrifuged at 10,000× *g* for 2 min at 4°C. The supernatant was transferred to a sterile tube and 500 μL of isopropanol was added. The mixture was centrifuged at 10,000× *g* for 2 min, after which the supernatant was removed. The pellet was washed with 10 μL of 70% cold ethanol and centrifuged at 8,000× *g* for 2 min. The pellets were air-dried, and the DNA was resuspended in 100 μL of DNase-free water. The 31 purified DNA samples were stored at −20°C until use.

### ERIC-PCR analysis

Because multiple colonies may have been selected from the same sample (Table 1), an ERIC-PCR analysis was performed on 14 isolates to identify if there were clones amongst the colonies. Genomic DNA was extracted as described above. The extracted DNA (100 ng) was used directly in a PCR reaction with previously described primers and conditions [21], conducted in a TGradient thermocycler (Biometra, Goettingen Germany). Twenty-five microlitre aliquots from each of the PCR amplifications were separated by electrophoresis for 2 h in 2% (m/v) agarose gels at 100 V, and then visualised by ethidium bromide staining. The DNA molecular weight marker VI (0.15–2.1 kb) (Roche Applied Science, Indianapolis, IN, USA) was used to estimate product sizes. A negative reaction control was included, and the *A. caviae* IIH47 strain was used as a positive control for each ERIC-PCR run.

**Table 1:** Description of vaginal lactobacilli as determined by culture- and PCR-based identification

**Isolate**	**Age of participant n = 21**	***Lactobacillus *group**	**Genetic ID**	**Nugent score**	**Co-infecting microorganism (s)**
Clinical isolates with *Lactobacillus* group n = 25					
003-051-PCP-a†	32	*acidophilus*	*L. gasseri*	N	
003-051-PCP-b			*L. gasseri*	N	
003-056-PAG-a‡	22		*L. gasseri*	N	Uu
003-056-PAG-b			*L. gasseri*	N	Uu
003-020-JPG†	26		*L. gasseri*	N	
003-022-KCA-a‡	23		*L. gasseri*	I	Uu + Ca
003-009-YTG†	27		*L. gasseri*	N	
003-028-SGT‡	48		*L. jensenii*	N	Uu
003-006-MAH†	22		*L. jensenii*	N	
003-022-KCA-b			*L. jensenii*	I	Uu + Ca
003-069-MFA-a†	20		*L. jensenii*	N	
003-052-LSC-a†	23		*L. crispatus*	N	
003-047-CMM†	36		*L. crispatus*	N	
003-015-PBS†	22		*L. crispatus*	N	
003-021-BVM-a‡	21	*reuteri*	*L. fermentum*	N	Uu
003-021-BVM-b			*L. fermentum*	N	Uu
003-069-MFA-b			*L. fermentum*	N	
003-069-MFA-c			*L. fermentum*	N	
003-017-LHH†	50		*L. fermentum*	N	
003-043-BNT‡	26		*L. fermentum*	N	Gv
001-JMB211‡	38	*casei*	*L. rhamnosus*	N	Ca
001-SMT40-a†¥	49		*L. rhamnosus*	N	
001-SMT40-b¥			*L. rhamnosus*	N	
001-SMT40-c			*L. rhamnosus*	N	
003-052-LSC-b		*buchneri*	*L. brevis*	N	
Strains without *Lactobacillus* group, n = 6					
003-025-COM†	38		*L* sp.	N	
003-002-PBT-a‡	25		E. faecalis*	I	Uu + Ct
003-002-PBT-b			E. faecalis*	I	Uu + Ct
003-036-MSH†	49		E. faecalis*	ND	
003-029-MNG‡	45		E. faecalis*	N	Uu
003-013-AMD‡	28		E. faecalis*	N	Uu
Reference strains, n = 3					
*L. brevis* 95b	NA	*buchneri*	*L. brevis*	NA	NA
*L. brevis* 95a	NA		*L. brevis*	NA	NA
*L. acidophilus* ENCB		*acidophilus*	*L. jensenii*	NA	NA

Normal = 0–3 (N), intermediate = 4–6 (I), Ct = *Chlamydia trachomatis*, Uu = *Ureaplasma urealyticum*, Gv = *Gardnerella vaginalis*, Ca = *Candida* spp., NA = not applicable, ND not determined. *presumptive identification. ^†^healthy, ^‡^unhealthy. ^¥^001-SMT40-a and 001-SMT-b are clones. 001-SMT40, 003-017-LHH and 003-036-MSH samples were from postmenopausal women.

### Amplification of 16S rRNA genes

DNA samples from the 31 isolates and three reference strains were subjected to PCR analysis. PCR was performed in a TGradient thermocycler (Biometra, Goettingen Germany), using primers that have been described previously [22]. The PCR amplifications were performed as follows: 2 μL of the PCR template was used in a 50 μL PCR mixture containing 1× PCR buffer with 2 mM MgCl_2_ (Invitrogen, São Paulo Brazil), 0.3 mM of each dNTP (Invitrogen), 0.2 μM of each primer, and 2.5 units of *Taq* DNA polymerase (Invitrogen). PCR amplification was conducted with the following temperature profile: an initial denaturation of 5 min at 94°C, 31 cycles of 30 s at 94°C, 60 s at 55°C, and 90 s at 72°C, followed by 5 min at 72°C. The PCR products were resolved by electrophoresis in a 1% (w/v) agarose gel and visualised by ethidium bromide staining.

### DNA sequencing

A total of 31 PCR products were purified using a PureLink Quick Gel Extraction Kit (Invitrogen, Carlsbad, CA, USA) according to the manufacturer’s instructions. The products were directly sequenced on an ABI-PRISM 310 Genetic Analyzer (Applied Biosystems, Foster City, CA, USA) using the forward and reverse primers used for PCR, according to the manufacturer’s instructions. Ambiguous and incorrectly called bases were manually corrected using Chromas Lite software, version 2.01 (Technelysium Pty Ltd.) and Seaview software version 4.3.3 [23]. To identify the isolates, the 31 sequences were compared to the V1–V3 regions of the lactobacilli 16S rRNA gene sequences available in the GenBank DNA database using the BLAST algorithm (http://www.ncbi.nih.gov). Sequences from the top BLAST hits were downloaded for further phylogenetic comparison, and were from: *L. iners* AY526083, *L. johnsonii* FJ542293, *L. gasseri* AB517146, *L. acidophilus* GQ202838, *L. ultunensis* AY253660, *L. gallinarum* EF412985, *L. helveticus* AB446394, *L. intestinalis* NR_025449, *L. ultunensis* AY253660, *L. gasseri* ATCC 33323 NR_041920, *L. jensenii* ATCC 25258 NR*_*025087 and *L. crispatus* ATCC 33820 NR_041800 from the *L. acidophilus* group. *L. mucosae* FJ751778, *L. gastricus* AY253658, *L. secaliphilus* AM279150, *L. fermentum* GQ922600, *L. ingluviei* NR_028810, *L. reuteri* GQ202839, *L. pontis* FJ749718, *L. antri* NR_027206, *L. vaginalis* AF243177 and *L. frumenti* NR_025371 from the *L. reuteri* group. *L. kefiri* FJ749410, *L. parabuchneri* AB429372, *L. hilgardii* FJ749461, *L. farraginis* AB262733, *L. hammesii* AB512777, *L. parabrevis* AB512779 and *L. brevis* M58810 from the *L. reuteri* group. *L. plantarum* GQ900607 from the *L. plantarum* group. *L. agilis* M58803, *L. salivarius* FJ751787 and *L. hayakitensis* AB267406 from the *L. salivarius* group. *L. casei* ATCC 393 NR_041893 and *L. rhamnosus* NM-945 HM218396 from the *L. casei* group. *E. faecalis* HNS5 FJ378702 was also included.

A multiple sequence alignment was performed using the program Clustal X, version 2.0 [24], and the resulting alignment was edited using SeaView [25]. A phylogenetic tree was constructed based on the sequence distances using the neighbour-joining (NJ) algorithm with the Tamura-Nei substitution model. The phylogenetic analyses were performed using Mega 4 [26]. The stability or accuracy of the inferred topology was assessed via a bootstrap analysis of 1,000 replicates. The identities of the sequences were determined based on the highest percentage (a minimum of 97%) of the total nucleotide match with sequences from GenBank [27,28].

## Results

In total, 31 isolates from 21 vaginal discharge samples from healthy (H; n = 12) and BV intermediate (unhealthy) (UH; n = 9) non-pregnant Mexican participants were phenotypically identified. All isolates were Gram-positive, non-motile, non-spore forming, catalase-negative, short or large rods, with morphology characteristic of *Lactobacillus* when grown on MRS medium. The quality and purity of genomic DNA from the 31 isolates and the three reference strains was sufficient to amplify the 16S rRNA gene, and partial 16S rRNA gene sequences were obtained for all samples. Because the first 510 bp of this gene include the variable regions V1–V3, which provide sufficient information for sequence analysis, only this region was considered in the *in vitro* analyses. To identify the isolates, a comparison of the sequences obtained in this study with those deposited in the GenBank database was performed using DNA sequence alignment and bioinformatics analysis. Sequences identified as *Lactobacillus*, specific species related to the vaginal habitat, and named and verified type strains deposited in the database at the time of retrieval were used in the analysis. Figure 1 shows the phylogenetic tree obtained from the sequence alignment.

The sequences of the 25 (25/31) *Lactobacillus* isolates were classified into the four groups of lactobacilli described by Ljungh and Wadström [29] as follows: 14 isolates (14/25) were classed as *L. acidophilus*, six isolates (6/25) were *L. reuteri*, four isolates (4/25) were *L. casei* and one isolate (1/25) was classed as *L. buchneri*. These results were similar to what has been reported in the literature. Surprisingly, five strains (5/31) were presumptively identified as *E. faecalis* by molecular characterisation, and one strain (1/31) could not be identified at the species level and was not clustered within any of the *Lactobacillus* groups proposed by Ljungh and Wadström. Using Nugent’s criteria, 26 strains were isolated from vaginal discharge that was scored as normal (26/31), four were obtained from samples scored as intermediate (4/31) and one sample was not evaluated using Nugent’s criteria (1/31).

Among the participants from which lactobacilli were isolated (n = 16), 13 were colonised by one *Lactobacillus* species, and three individuals were colonised by two different lactobacilli (Table 1). The remaining five samples contained *Enterococcus* and *Lactobacillus* species. Because more than one colony could have been chosen from the same sample, an ERIC-PCR analysis was performed (n = 14). The analysis showed that only two isolates from one sample were clones (SMT40). In contrast, the profile analysis indicated the occurrence of intraspecific variability among the *L. gasseri* and *L. fermentum* species. For example, different ERIC-PCR profiles between isolates from the same sample were obtained, including PCP-a vs. PCP-b, PAG-a vs. PAG-b, BVM-a vs. BVM-b, and MFA-b vs. MFA-c, and between strains isolated from different samples, including PCP (a or b) vs. PAG (a or b) and BVM (a or b) vs. MFA (b or c).

**Figure 2:**
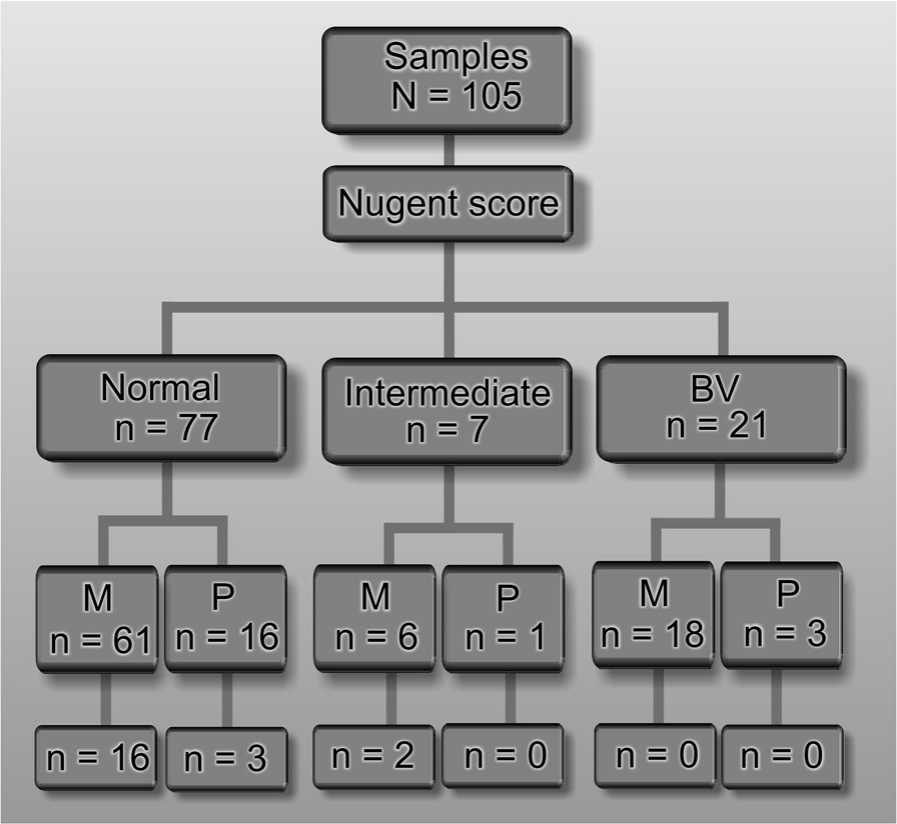
**Flow chart of *Lactobacillus *spp. isolation in the different participant groups.** Classification was performed using Nugent’s score (normal (N), score 0–3, intermediate (I), score 4–6; bacterial vaginosis (BV), score ≥7) and hormonal status of the participants (menarche, M, and postmenopausal, P).

Because other microorganisms were identified in a previous study that had 105 samples [17], the incidence of co-infection could be analysed. Co-infection with lactobacilli and *Chlamydia trachomatis*, *Ureaplasma urealyticum*, *Gardnerella vaginalis* and *Candida albicans* was observed. *L. gasseri* was the most frequently isolated species (n = 7; H = 4, UH = 3), followed by *L. fermentum* (n = 6; H = 3, UH = 3), *L. jensenii* (n = 4; H = 2, UH = 2), *L. crispatus* (n = 3; H = 3, UH = 0), *L. rhamnosum* (n = 3; H = 2, UH = 1) and *L. brevis* (n = 1; H = 1, UH = 0). Because of the low number of isolates, it was not possible to conclusively determine whether a particular species was associated with a healthy or unhealthy status. However, in general, *L. gasseri* (4/7) and *L. crispatus* (3/3) were more frequently detected in the normal samples than in the “unhealthy” group (Table 1). When the interpretation of the smears according to Nugent’s criteria was considered, the *Lactobacillus* isolates were classified into only two of the three possible groups (normal and intermediate); accordingly, *Lactobacillus* was not isolated from the samples with BV, although one isolate was co-isolated with *G. vaginalis* (Figure 2).

## Discussion

Lactobacilli are an important part of the vaginal microbiota. The presence of lactic acid, H_2_O_2 _and other by-products of these bacteria are beneficial in controlling the overgrowth of potentially pathogenic bacteria (i.e., bacterial vaginosis). For example, Antonio *et al.*[30] reported that women who were not colonised with H_2_O_2_-producing lactobacilli, such as *L. crispatus, L. iners, L. jensenii, L. gasseri* and *L. vaginalis*, were 15 times more likely to have bacterial vaginosis than woman who were colonised by these strains. Despite their importance to women’s health, vaginal lactobacilli have not been extensively studied in the Mexican population. Historically, *L. acidophilus* was considered to be the dominant species in the human vagina. It is now known that the group of organisms previously known as *L. acidophilus* is highly heterogeneous, and includes at least six separate species [29,31]. In addition, it has been shown that the majority of vaginal *Lactobacillus* strains from women of geographically separated countries belong to the four species *L. iners*, *L. crispatus*, *L. gasseri* and *L. jensenii*, indicating a high degree of species consistency in vaginal lactobacilli among women worldwide [32]. The identity of the vaginal lactobacilli in Mexican women has not been well-studied, and the majority of published papers have used phenotypic approaches. Therefore, we based our approach on 16S rRNA gene sequencing and ERIC-PCR analysis.

The isolation of lactobacilli in the present study was performed using a general selective medium, and relatively few strains were isolated. This result is consistent with other reports, which indicate that lactobacilli may go undetected in the laboratory because their growth requires unique media and an extended incubation time [31]. In 2001, Angeles-Lopez *et al*. [2] reported the isolation of lactobacilli in only 87 of 156 samples inoculated on MRS agar. In addition, it has been reported that *L. iners*, a species belonging to the *L. acidophilus* group, does not grow on MRS agar. Therefore, in the present study and other studies using MRS, this species was not evaluated and has been misidentified [33]. Other authors have noted that even after recovery, strain misidentification can occur because the strains morphologically resemble those of other genera, including *Corynebacterium*, *Clostridium* and *Streptococcus*[31]. Given the difficulty in isolating these bacteria, and the possibility that they may often be misidentified, culture-independent genetic approaches are now preferred over culture-dependent methods [34]. Moreover, several types of vaginal microbiota exist in healthy women. Although *Lactobacillus* is often the predominant genus, the vaginal microbiota also includes a diverse assemblage of anaerobic microorganisms, which likely occur within the mestizo and Mexican populations.

When strains can be isolated, their identification can be aided by molecular techniques to distinguish between closely related species within the *Lactobacillus* genus, which can be impossible by phenotypic methods alone. Although a large number of molecular methodologies are currently available to study these bacteria, some of the techniques, such as PCR, denaturing gradient gel electrophoresis (DGGE) and thermal gradient gel electrophoresis (TGGE) [9,34-37], still require improvement, especially with regard to the sensitivity, cost and quantitative power. Among all available molecular techniques, 16S rRNA sequencing analysis has been accepted as the most reliable method. Therefore, this method was used in the present work to provide the first genetic identification of the indigenous microbiota of the vaginal cavity of non-pregnant Mexican women.

The majority of the species detected in the Mexican population in the present study belong to the *L. acidophilus* group, although strains of the *L. reuteri*, *L*. *casei* and *L*. *buchneri* groups have also been identified [29]. The majority of the lactobacilli found in the vaginal communities were phylogenetically related to *L. gasseri, L. fermentum, L. rhamnosus, L. jensenii, L. crispatus, L. fornicalis* or *L. brevis*, which is at least partially consistent with previous reports on the species distribution in other countries [6,17,32,38]. However, other lactobacilli species have been described at a lower frequency, including *L. vaginalis*, *L. fermentum*, *L. mucosae*, *L. paracasei* and *L. rhamnosus*, in reports that have indicated the variability among women of specific regions [6,32,39,40]. This trend is consistent with our results, in which *L. fermentum, L. rhamnosus* and *L. brevis* were also identified. These findings are not surprising since it has become increasingly apparent in recent years that ethnicity can affect the number and type of organisms present in the vaginal cavity [17,18,37].

A previous study published by Hernández-Rodríguez *et al.*[9] did not identify *L. crispatus* using a culture-independent method (DGGE-PCR) in samples from pregnant Mexican women, which is in contrast with our study, in which this species was isolated, albeit at a low frequency [17,18,38,40].

The absence of *L. iners* isolates in our study is significant because it is one of the dominant species reported worldwide. The lack of detection of *L. iners* could be attributed to the limitations of the methodology used in the current analysis, and must be confirmed in future studies without these limitations (e.g. DGGE of PCR-amplified 16S rRNA fragments can overcome the limitation of traditional cultivation techniques to retrieve the vaginal econiche diversity).

Correct species identification is dependent on the reliability of the reference strains and accuracy of the database used. Because many reference strains were previously characterised using non-genetic methods, it is possible that they were misidentified and, thus, the reference strains themselves may be unreliable [32]. For example, the reference strain previously identified as *L. acidophilus* should be re-labelled as *L. jensenii* based on the genetic data obtained in the present study (Table 1). Both species belong to the *L. acidophilus* group and are genetically related.

The majority of the strains isolated in this study were identified as species of the *L. acidophilus* group. Because *Lactobacillus* species are considered to be critical for protection against pathogens in the female genital tract, this set of strains could be useful in future studies on probiotic properties [29], especially given the potential differences in the protective capabilities of vaginal *Lactobacillus* species.

The intraspecific variability among the *L. gasseri* and *L. fermentum* species, detected by ERIC-PCR analysis, concurs with a previous report by Stephensen *et al*. [41]. This previous study showed that ERIC-PCR analysis was capable of typing *Lactobacillus* isolates at the strain level.

The main limitations of this study were the small sample size and problems associated with culture-dependent methods. In addition, because of the small number of isolates, it was not possible to correlate the observed species with a healthy or unhealthy (BV) status. Our results should be corroborated using a larger cohort and culture-independent methods (DGGE or sequencing of cloned 16S rRNA molecules) to describe the relative abundance of the species described herein. Despite these limitations, the results of this study concur with previously published findings showing that *L. crispatus, L. gasseri* and *L. jensenii* are consistently present in the healthy vaginal ecosystem, and provide additional information regarding the Mexican (Hispanic-mestizo) non-pregnant population [6,17,32,38,40,42]. Additional species or phylotypes not common in other countries were found in this study, which furthers our understanding of vaginal colonisation by lactobacilli, and the *Lactobacillus* species diversity in vaginal communities in the mestizo population.

## Conclusions

Accurate phenotypic identification of species of the genus *Lactobacillus* is difficult. The use of molecular techniques in combination with culture-based methods adds greatly to our understanding of the normal microbiota of a particular environment. The Mexican (Hispanic-mestizo) non-pregnant population is colonized mainly by *L. acidophilus* group lactobacilli. The majority of the lactobacilli identified in the Mexican vaginal communities were *L. gasseri*, *L. fermentum*, *L. rhamnosus*, *L. jensenii*, *L. crispatus* and *L. brevis*. Because the dominant *Lactobacillus* species may differ depending on race or geography, the ability to identify lactobacilli at the species level should enable us to better understand the roles of the various *Lactobacillus* species. Culture-independent techniques must be used in future analyses to overcome the difficulty in isolating these bacteria and to prevent misidentification. Molecular methods can also provide a wider description of microbial communities, and measure prevalence, diversity and abundance of vaginal microbiota, which also includes a diverse assemblage of anaerobic microorganisms or more fastidious lactic acid-producing bacteria.

## Competing interests

The authors declare that they have no competing interests.

## Authors’ contributions

MDMP carried out the molecular genetic studies, participated in the sequence alignment and helped to draft the manuscript. GCE helped to draft the manuscript and participated in the data analysis. MGAA conceived of the study, participated in its design and coordination, assessed the data and drafted the manuscript. All authors read and approved the final manuscript.

## Pre-publication history

The pre-publication history for this paper can be accessed here:

http://www.biomedcentral.com/1471-2334/13/189/prepub
